# From fertility intentions to contraceptive behaviour: evidence among women with children in Malawi

**DOI:** 10.3389/frph.2026.1780852

**Published:** 2026-04-22

**Authors:** Redson Mwandama, Sydney Nkhoma, Margubur Rahaman, Christopher Chombo, Gladson Andrew Chipala

**Affiliations:** 1 School of Law, Economics, and Government, Department of Economics, University of Malawi, Zomba, Malawi; 2 Department of Agricultural and Applied Economics, Lilongwe University of Agriculture and Natural Resources, Lilongwe, Malawi; 3 Research Consultant, Govind Ballabh Pant Social Science Institute, Prayagraj, India

**Keywords:** contraceptive use, fertility intentions, limiting demand, Malawi, sub-Saharan Africa

## Abstract

**Background:**

Although fertility has declined in Malawi, a substantial gap persists between women's fertility-limiting intentions and contraceptive behaviour. Previous studies often include childless women, potentially obscuring patterns among women who have already entered parenthood. This study examines fertility preferences and contraceptive behaviour among in-union women with at least one living child in Malawi.

**Methods:**

We analysed cross-sectional data from the 2015–16 Malawi Demographic and Health Survey. The fertility intention analysis included 15,866 married or in-union women aged 15–49 years with at least one living child. Multinomial logistic regression models were used to estimate adjusted average marginal effects (AMEs) for wanting another child and being undecided (reference: wants no more children). A sub-sample of women with limiting demand (*n* = 5,358) was analysed to examine modern and traditional contraceptive use using multinomial logistic regression. All analyses accounted for complex survey design.

**Results:**

Overall, 49.8% of women reported wanting no more children, while 44.7% desired additional children. The likelihood of wanting another child declined markedly with age (AME = −0.308 among women aged 35–49 years) and parity (AME = −0.504 among women with four or more children). Muslim women were more likely to desire additional children (AME = 0.053). Among women with limiting demand, 72.4% used modern contraception, 25.4% used no method, and 2.2% relied on traditional methods. Modern method use was positively associated with higher parity, primary and secondary education, employment, urban residence, and residence in the Central and Southern regions, while older age and Muslim religion were negatively associated. Side effects (37.4%) were the leading reason for discontinuation of modern methods.

**Conclusions:**

In Malawi, fertility preferences strongly reflect parity and age, yet a substantial proportion of women with limiting demand remain non-users of contraception. Reducing unmet need requires strengthening access, method continuation support, and regionally responsive family planning strategies.

## Introduction

Fertility preferences and contraceptive behaviour are central indicators of sexual and reproductive health and reproductive rights, reflecting individuals' capacity to make informed decisions regarding the timing and number of births (1). Persistently high fertility combined with low contraceptive use signals reproductive vulnerability, constrained autonomy, and inadequate access to quality reproductive health services among women (2, 3). While the global demographic transition has progressed substantially, sub-Saharan Africa continues to lag behind other regions, characterised by elevated fertility and mortality rates (4). Within this regional context, Malawi remains a critical case where fertility levels are declining but remain high, and contraceptive use continues to exhibit marked social and geographic inequalities. Evidence from successive Malawi Demographic and Health Surveys (MDHS) shows that total fertility rate (TFR) declined from more than six births per woman in the early 1990s to approximately 4.4 births by 2015–16 in Malawi (5).

Despite this progress, fertility remains considerably above global averages and varies sharply by residence, education, and socioeconomic status. Although modern contraceptive use has expanded nationally, uptake remains uneven, particularly among adolescents, rural residents, and women with lower levels of education (6). These persistent disparities highlight the need for analytical approaches that move beyond descriptive trends to examine how fertility preferences, contraceptive behaviour, and access to reproductive health services interact to shape reproductive outcomes. Recent empirical research increasingly adopts integrated frameworks that jointly consider preferences, access, and social context (7). Large-scale DHS-based studies demonstrate that women's fertility preferences often diverge from contraceptive behaviour; many women intending to limit or space births do not use contraception, suggesting that constraints beyond individual preference play a critical role (8–10).

Fertility preferences are also dynamic rather than fixed. Longitudinal studies indicate that preferences are particularly fluid among younger women and in contexts of partner disagreement (11, 12). External shocks, including the COVID-19 pandemic and climate-related disruptions, further heighten uncertainty, leading to delayed childbearing and short-term changes in contraceptive behaviour (13, 14). Beyond individual preferences, social and interpersonal factors strongly influence contraceptive use. Across African settings, concerns regarding side effects, partner opposition, and restrictive community norms consistently emerge as key barriers, even where services are physically available (15, 16). In Malawi, while modern contraceptive prevalence has increased, inequalities persist by age, education, and residence (17, 18). Household wealth quintile and spousal communication are strong predictors of use, whereas adolescents experience particularly high unmet need. Partner migration further shapes fertility and contraceptive behaviour in complex ways (19). Qualitative studies underscore that reproductive decisions are embedded within networks of partners, peers, and kin, often characterised by misinformation and gendered power imbalances (20, 21). These findings emphasise that meaningful access extends beyond service availability to include privacy, respectful care, and social legitimacy.

Despite substantial evidence on fertility preferences and contraceptive use, existing studies have largely overlooked the specific patterns and predictors of fertility intentions among women who have at least one living child and whose fertility is not permanently limited through sterilization female or partner vasectomy (22). Prior research often includes childless women in analyses of fertility intentions, which may introduce measurement bias. Women without children are more likely to report a desire for more children by default, potentially underestimating the true proportion of women who intend to stop childbearing. To address this limitation, the present study focuses on women who have already achieved at least one birth, thereby providing a more precise assessment of fertility intentions beyond the normative expectation of childbearing. Furthermore, previous studies frequently examined contraceptive use in the general population without distinguishing women who have expressed a demand for limiting childbearing in Malawi (23). From a demographic and public health perspective, understanding contraceptive behaviour specifically among women who wish to stop childbearing is critical for reducing unintended pregnancies and, ultimately, high total fertility rates in Malawi (24). Accordingly, the second objective of this study investigates the patterns and predictors of contraceptive method use among women with limiting demand. In addition, among women with limiting demand who were not currently using contraception, this study examines reasons for last method discontinuation. Identifying these reasons provides important insights into method-related, service-related, and contextual barriers that may hinder sustained contraceptive use.

By integrating fertility intentions, contraceptive behaviour, and discontinuation dynamics within a clearly defined at-risk population, this study offers a more refined and policy-relevant contribution to the literature. The findings are expected to inform targeted family planning strategies by identifying subgroups with persistent limiting demand but inconsistent contraceptive use. Moreover, the evidence can support programmatic efforts to improve method continuation, strengthen counselling on side effects, and enhance service delivery to reduce unintended fertility and support progress toward national reproductive health goals.

## Methodology

### Study design and data source

This study used a cross-sectional analytical design based on secondary data from the 2015–16 Malawi Demographic and Health Survey (MDHS). The analysis relied on the Women's Individual Recode (IR) file, which contains detailed information collected from women aged 15–49 years on fertility and reproductive histories, contraceptive knowledge and use, maternal and child health, sexual and reproductive health behaviours, marriage and sexual activity, and women's status indicators. The IR dataset also includes socio-demographic and economic characteristics such as age, education, household wealth, residence, region, religion, and employment. The MDHS employed a stratified two-stage cluster sampling design to ensure national representativeness across urban and rural areas and Malawi's administrative regions, with survey weights and design variables applied in all analyses to account for the complex sampling design. The details of the MDHS sampling design available in the published report (25).

### Analytical sample

**Figure 1:**
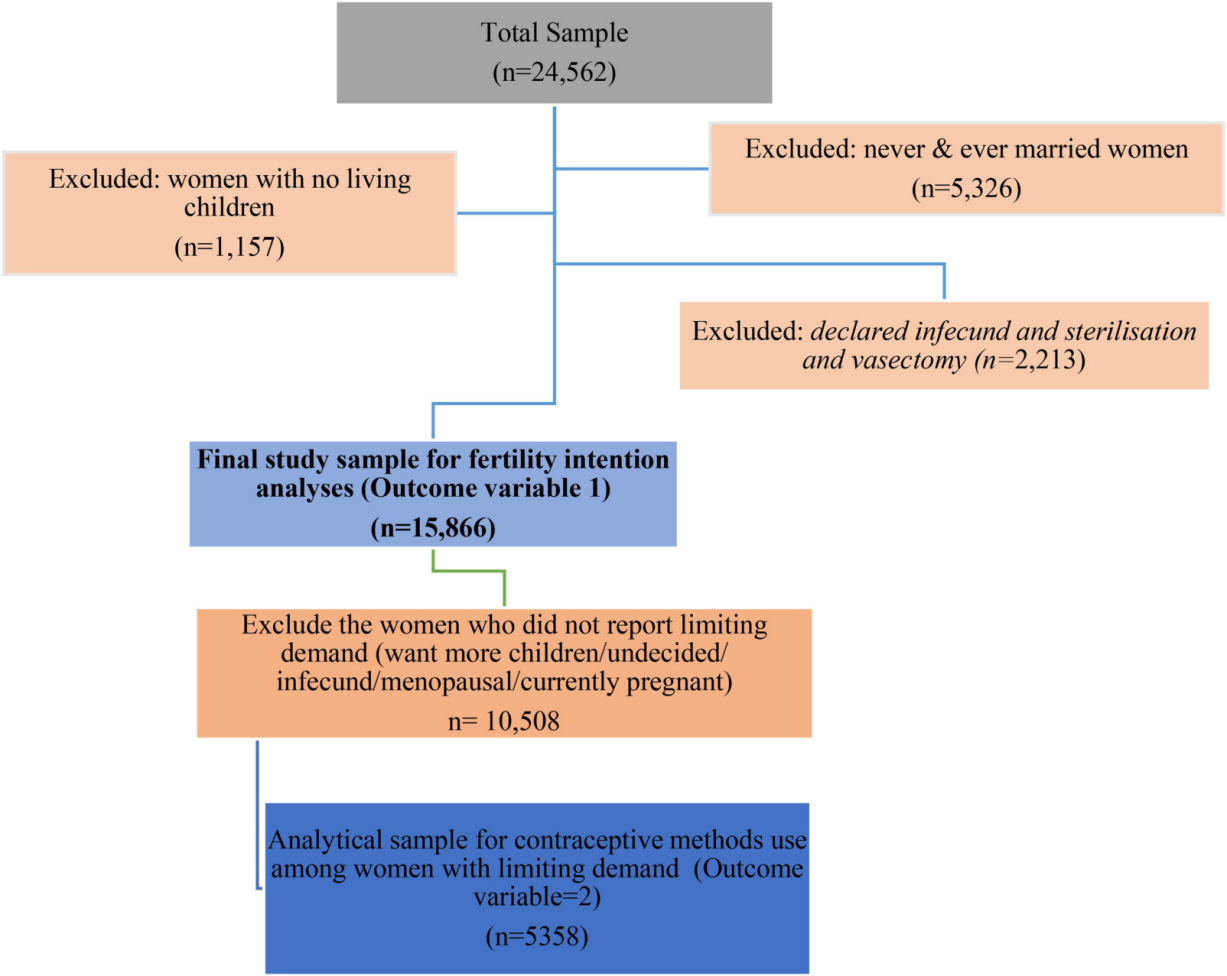
Flow chart of selecting sample of present study analyses, MDHS 2015–16.

Although the MDHS IR dataset included 24,562 women aged 15–49 years (25), the present study included 15,866 women who were currently married and had at least one living child without a history of sterilization (female or partner). The analytical sample was restricted to currently married or in-union women with at least one living child, as fertility preferences and contraceptive decision-making are most meaningful among women exposed to regular pregnancy risk and who have entered parenthood (26). This selected sample was used to examine fertility intention patterns among the study women. Further, only women with limiting family planning intentions were included to examine the patterns and predictors of use of modern and traditional contraceptive methods. To identify women with limiting demand, we followed the revised definition of family planning demand (27). As a result, a weighted sample of 5,358 women was included after excluding women with no limiting fertility demand, those who were undecided, infecund, menopausal, or currently pregnant during the analysis of contraceptive method use (28). The details of the analytical sample selection are presented in Figure 1.

### Outcome variables

#### Fertility intentions

Following the previous literature (29), fertility intentions were measured using the question: “Would you like to have (a/another) child, or would you prefer not to have any (more) children?” Responses were categorized into three mutually exclusive categories: Want more (a/another) children (coded as 1), Undecided (coded as 2), and want no more children (coded as 0).

### Use of contraceptive methods

Based on previous studies (30), use of contraceptive methods was categorized into three mutually exclusive groups: Not use any methods Non-use (coded as 0), use of traditional contraception (coded as 1), and use of modern contraception (coded as 2). Modern contraceptive methods included pill, intrauterine device (IUD), injectables, male condom, implants/Norplant, lactational amenorrhea method (LAM), and female condom. Traditional methods included periodic abstinence (rhythm method), withdrawal, other traditional methods, and the Standard Days Method. Women who reported not using any contraceptive method were classified as non-users.

### Explanatory variables

Based on prior literature (31, 32), a range of socio-demographic, reproductive, economic, and informational factors were selected as explanatory variables. In particular, age (15–19, 20–24, 25–34, and 35–49 years), number of living children (one, two, three, four or more), sex composition of living children (only daughters, only sons, and both sexes), household wealth quintile (poorest, poorer, middle, richer, richest) and employment status (no, yes) were selected as demographic and economic factors. In DHS, household wealth quintiles were constructed through principal component analysis (PCA) of household asset ownership, housing characteristics (e.g., flooring, roofing materials), and access to water, sanitation, and other durable goods. The resulting composite score is standardized and households are ranked and divided into five equal quintiles (poorest to richest) within each survey to represent relative socioeconomic status.

In addition, education level (none, primary, secondary, and tertiary), exposure to family planning information (no, yes), decision-making autonomy (no, yes), and religion (Christian, Muslim, and Other) were included as socio-cultural factors. Geographical factors included place of residence (urban/rural) and region (Northern, Central, and Southern). Exposure to family planning information was coded as “yes” if women reported receiving family planning messages from at least one media source (radio, television, newspapers/magazines, or mobile text messages), and “no” otherwise. Women's decision-making autonomy was classified as “yes” if they reported participating (alone or jointly with a partner) in any of the following domains: own health care, major household purchases, or visits to relatives, and “no” if participation was absent in all domains.

### Statistical analysis

Univariate, bivariate, and multivariate analyses were applied to accomplish the study objectives. Univariate analyses presented the socio-demographic and economic characteristics of the study population along with their fertility intentions and contraceptive use. Bivariate analyses with Pearson's chi-square tests showed variation in fertility intentions and contraceptive use among the study population by their socio-demographic, economic, and geographic characteristics. Univariate distributions were presented as column percentages, whereas bivariate distributions were presented as row percentages. All these descriptive analyses were performed using DHS sampling weights.

Multivariable multinomial logistic regression models were fitted to examine the associations between selected explanatory variables and the two categorical outcomes (fertility intentions and use of contraceptive methods). For the multinomial logistic regression analysis, “Want no more children” (coded as 0) was considered as the reference category to estimate adjusted average marginal effects (AMEs) for the desire for more children and undecided fertility intention outcomes relative to wanting no more children. Adjusted average marginal effects (AMEs) were estimated to facilitate interpretation of the magnitude and direction of associations. However, a multivariable multinomial logistic regression model was also applied to estimate the adjusted beta coefficients for the use of modern and traditional methods relative to not using any contraceptive methods among the study women.

The general form of the multinomial logit model is:

$$\log\left(\frac{P(Y=j)}{P(Y=0)}\right)=\beta_{0j}+\beta_{1j}X_{1}+\ldots +\beta_{pj}X_{p}$$

where *P*(*Y* = *j*) represents the probability of outcome category *j*, and *P*(*Y* = 0) is the probability of the reference category. Before regression analyses, multicollinearity was assessed by performing variance inflation factor (VIF) diagnostics for both models. No evidence of problematic multicollinearity was observed among the explanatory variables (Supplementary Table S1). Regression model fit was evaluated using McFadden's pseudo-R², indicating a good model fit. Standard errors (SEs) were adjusted for clustering and stratification using survey linearization methods. AMEs and beta coefficients were computed with MDHS survey design correction using the “svy” command in STATA (version 19.5) software. Exploratory interactions (age×parity; education×residence) were tested for both outcome variables and presented in the Supplementary File. Survey weights (v005/1,000,000) and design variables were applied in all analyses to account for the complex sampling design.

### Ethical considerations

This study uses secondary, de-identified data from the 2015–16 Malawi Demographic and Health Survey. The survey received ethical approval from the Malawi National Health Sciences Research Committee and relevant institutional review boards, and informed consent was obtained from all participants. Data access was granted by the DHS Program. As the analysis used publicly available, anonymized data, additional institutional ethical approval was not required. All analyses adhered to standard principles of confidentiality and responsible data use.

## Results

### Study population characteristics

A total of 15,866 weighted women were included in the analysis (Table 1). The largest proportion were aged 25–34 years (42.6%), followed by 35–49 years (28.3%) and 20–24 years (23.7%), while adolescents aged 15–19 years comprised 5.4% of the sample. More than one-third of women had four or more living children (36%), whereas 22.2% had one child, 22.1% had two children, and 19.8% had three children. The majority (59.1%) had children of both sexes, while 20.5% had only girls and 20.4% had only boys. Most women had primary education (64%), followed by secondary education (19.7%); 14.0% had no formal education and 2.3% had tertiary education. The majority were Christian (85.2%), followed by Muslim (14.1%). Approximately one-fifth belonged to the poorest wealth quintile (22%). More than two-thirds of women were working (68.1%), and 38.2% reported decision-making autonomy. Over half (55.9%) had not been exposed to family planning messages. The majority resided in rural areas (83.7%), and nearly half were from the Southern region (47.8%), followed by the Central (40.2%) and Northern (12.1%) regions.

**Table 1:** Background characteristics of the study women (15-49 years), Malawi, MDHS 2015–16 (*N* = 15,866).

Characteristic	Weighted sample (n)	Percent (%)
Age group (years)		
15–19	862	5.4
20–24	3,758	23.7
25–34	6,760	42.6
35–49	4,486	28.3
Number of living children		
1 child	3,525	22.2
2 children	3,500	22.1
3 children	3,133	19.8
4 + children	5,707	36
Sex composition of living children		
Only girls	3,257	20.5
Only boys	3,232	20.4
Both sexes	9,377	59.1
Education Level		
No education	2,225	14
Primary	10,156	64
Secondary	3,127	19.7
Tertiary	358	2.3
Religion		
Christian	13,517	85.2
Muslim	2,234	14.1
Other	115	0.7
Wealth quintile		
Poorest	3,486	22
Poorer	3,270	20.6
Middle	3,033	19.1
Richer	2,862	18
Richest	3,214	20.3
Employment status		
Not working	5,055	31.9
Working	10,811	68.1
Decision-making autonomy		
Not autonomous	9,812	61.9
Autonomous	6,053	38.2
Family planning message exposure		
No	8,872	55.9
Yes	6,994	44.1
Place of residence		
Rural	2,579	16.3
Urban	13,286	83.7
Region		
Northern	1,914	12.1
Central	6,369	40.2
Southern	7,582	47.8
Total	15,866	100.0

All estimations are based on MDHS survey-weight.

### Bivariate results of fertility preference

Overall, 44.7% of women aged 15–49 years expressed a desire for more children (Table 2). The desire for additional children was strongly patterned by age and parity (*p* < 0.001). It was highest among adolescents aged 15–19 years (84.2%) and declined markedly with increasing age to 11.7% among women aged 35–49 years. Similarly, 82.1% of women with one child wanted another child compared with only 14.5% among those with four or more children. Women with only girls (67.2%) or only boys (68.7%) were substantially more likely to want another child than those with children of both sexes (28.6%) (*p* < 0.001). A relatively higher preference for more children was also observed among women in poorer households (50.1%), those with secondary education (51.5%), Muslims (47.3%), and women exposed to family planning messages (46.2%). Whereas, nearly half of the women (49.8%) reported wanting no more children (Table 2). The preference to limiting childbearing increased sharply with age, from 11.6% among adolescents to 84.2% among women aged 35–49 years (*p* < 0.001). It was highest among women with four or more children (80.7%) and among those with children of both sexes (65.8%). Women with no education (64.1%), those in the richest wealth quintile (53.0%), working women (51.9%), and Christians (50.6%) were also more likely to desire no further children. A small proportion (5.5%) were undecided about their fertility desire (Table 2). Indecision was relatively higher among women aged 25–34 years (6.2%), those with two or three children (7.1% and 6.4%, respectively), Muslims (8.3%), not working women (8.4%), and women residing in the Northern region (10.2%).

**Table 2:** Bivariate distribution of fertility preference among women aged 15–49 years by background characteristics, Malawi DHS 2015–16 (*N* = 15,866).

Characteristic	Weighted sample (n)	Wants More Children (%)	Wants No More Children (%)	Undecided (%)	*p*-value
Age group (years)					
15–19	862	84.2	11.6	4.1	
20–24	3,758	74.8	19.1	6.1	<0.001
25–34	6,760	44.8	49.0	6.2	
35–49	4,486	11.7	84.2	4.2	
Number of living children					<0.001
1 child	3,525	82.1	13.8	4.2	
2 children	3,500	59.3	33.6	7.1	
3 children	3,133	41.2	52.4	6.4	
4 + children	5,707	14.5	80.7	4.9	
Sex composition of living children					
Only girls	3,257	67.2	26.9	6.0	
Only boys	3,232	68.7	26.6	4.7	<0.001
Both sexes	9,377	28.6	65.8	5.6	
Education Level					
No education	2,225	29.7	64.1	6.2	
Primary	10,156	45.7	49	5.3	<0.001
Secondary	3,127	51.5	42.8	5.7	
Tertiary	358	48	45.6	6.4	
Religion					
Christian	13,517	44.4	50.6	5.1	
Muslim	2,234	47.3	44.4	8.3	<0.001
None/Other	115	31.0	66.3	2.7	
Household Wealth Quintile					
Poorest	3,486	44.6	49.5	5.9	
Poorer	3,270	50.1	44.9	5.0	
Middle	3,033	44.3	49.8	5.9	<0.001
Richer	2,862	42.2	52.4	5.4	
Richest	3,214	41.8	53.0	5.3	
Employment Status					
Not working	5,055	46.1	45.4	8.4	<0.001
Working	10,811	44.0	51.9	4.1	
Decision-making autonomy					
Not autonomous	9,812	44.1	50.1	5.8	0.021
Autonomous	6,053	45.6	49.4	5.0	
Family planning message exposure					
No exposure	8,872	43.5	50.7	5.9	0.003
Any media exposure	6,994	46.2	48.8	5.0	
Place of Residence					
Rural	2,579	42.9	52.5	4.6	0.07
Urban	13,286	45.0	49.3	5.7	
Region					
Northern	1,914	44.2	45.7	10.2	<0.001
Central	6,369	47	49.4	3.7	
Southern	7,582	42.9	51.3	5.9	
Total	15,866	44.7	49.8	5.5	

Values are MDHS weighted sample row percentages. *p*-values were based on Pearson chi-square tests.

### Predictors of desire for more children

The desire for additional children was significantly linked with age and number of living children (Table 3). Compared with adolescents aged 15–19 years, women aged 25–34 years were significantly less likely to want more children, with substantial lower AME found among women aged 35–49 years (AME = −0.31, *p* < 0.001). Relative to women with one child, those with two children were 19.6 percentage points less likely to desire more children, and the AME increased to 50.4 percentage points among women with four or more children (*p* < 0.001). Sex composition of the children was significant predictor of fertility intensions. Women with children of both sexes were less likely to desire additional children compared with those who had only daughters (AME = −0.03, *p* < 0.05). Educational attainment was inversely associated with desire more children. The AME of desire of more children found to be negative among women with tertiary education (AME = −0.07, *p* < 0.05) compared to women with no education counterparts. Muslim women were significantly more likely than Christian women to express a desire for additional children (AME = 0.053, *p* < 0.001). Household wealth quintile and place of residence were also significant predictors of desire of more children. Compared with women in the poorest quintile, those in the poorer quintile were more likely to want more children (AME = 0.04, *p* < 0.01). Urban women were less likely desired more children than rural women counterparts (AME = −0.05, *p* < 0.001). Employment status (AME = 0.033, *p* < 0.001) and decision-making autonomy (AME = 0.02, *p* < 0.05) were positively associated with the desire for more children. Interaction effects indicate that increasing number of living children across older age groups is associated with lower probability of wanting another child and being undecided, with particularly strong negative marginal effects among women aged 35–49 years (Supplementary Table S2).

**Table 3:** Average marginal effects (Ames) from multinomial logistic regression examining predictors of wanting another child or being undecided (reference: wants no more children) among women aged 15–49 years in Malawi, DHS 2015–16 (*N* = 15,866).

Variables	Wants another child AME (SE)	Undecided AME (SE)
Age group		
15–19	0.000 (ref.)	0.000 (ref.)
20–24	0.006 (0.027)	0.009 (0.014)
25–34	−0.057** (0.029)	−0.002 (0.015)
35–49	−0.308*** (0.031)	−0.012 (0.016)
Number of living children		
1	0.000 (ref.)	0.000 (ref.)
2	−0.196*** (0.017)	0.027*** (0.008)
3	−0.313*** (0.021)	0.023** (0.009)
4+	−0.504*** (0.022)	0.018* (0.010)
Sex composition of children		
Girls only	0.000 (ref.)	0.000 (ref.)
Only boys	0.014 (0.013)	−0.014* (0.008)
Both sexes	−0.032** (0.013)	−0.009 (0.008)
Education level		
No education	0.000 (ref.)	0.000 (ref.)
Primary	−0.006 (0.013)	−0.011 (0.007)
Secondary	−0.031* (0.016)	−0.000 (0.009)
Tertiary	−0.069** (0.033)	0.039 (0.030)
Religion		
Christian	0.000 (ref.)	0.000 (ref.)
Muslim	0.053*** (0.012)	0.028*** (0.008)
None/Other	−0.043 (0.052)	−0.021 (0.017)
Household wealth quintile		
Poorest	0.000 (ref.)	0.000 (ref.)
Poorer	0.035*** (0.012)	−0.009 (0.007)
Middle	0.017 (0.012)	−0.000 (0.007)
Richer	0.010 (0.013)	−0.008 (0.007)
Richest	−0.010 (0.016)	−0.004 (0.009)
Employment status		
Not working	0.000 (ref.)	0.000 (ref.)
Working	0.033*** (0.009)	−0.036*** (0.005)
Decision-making autonomy		
Not autonomous	0.000 (ref.)	0.000 (ref.)
Autonomous	0.018** (0.008)	−0.006 (0.004)
Family planning message exposure		
No	0.000 (ref.)	0.000 (ref.)
Yes	0.012 (0.009)	−0.006 (0.005)
Place of residence		
Rural	0.000 (ref.)	0.000 (ref.)
Urban	−0.054*** (0.014)	−0.015** (0.007)
Region		
Northern	0.000 (ref.)	0.000 (ref.)
Central	−0.003 (0.012)	−0.064*** (0.009)
Southern	−0.017 (0.011)	−0.051*** (0.009)

Model statistics: Log-Likelihood=−11,009.232; Wald *χ*^2^(46) = 3,074.19; Prob > *χ*^2^ = 0.000. reference outcome category was “Want no more children”. ref.: reference category; AME: average marginal effects. AMEs are computed with MDHS survey design correction.

Significance levels: *p < 0.05; **p < 0.01; **p < 0.001.

### Predictors of undecided fertility intentions

The number of living children was positively associated with fertility decision uncertainty (Table 3). Compared with women who had one child, those with two (AME = 0.027, *p* < 0.001), three (AME = 0.023, *p* < 0.01), and four or more children (AME = 0.018, *p* < 0.05) were more likely to be undecided about their fertility intentions. Employment status was found to be negatively associated with the likelihood of being undecided (AME = −0.036, *p* < 0.001). Muslim women were more likely than Christian women to report uncertainty (AME = 0.028, *p* < 0.001). Urban residence was associated with lower uncertainty compared with rural residence (AME = −0.015, *p* < 0.01). Regional differences were pronounced, with women in the Central and Southern regions significantly less likely to report indecision than those in the Northern region.

### Contraceptive use among women with fertility limiting demand

Among women with fertility limiting demand, 72.4% were reported use of modern methods, 25.4% were not using any method, and 2.2% relied on traditional methods (Table 4). Use of modern methods was high among women aged 20–24 years (85.2%), followed by women aged 15–19 years (83.1%). The proportion using modern methods was substantially higher among women with two children (78.7%), followed by women with three children (76.7%). Likewise, a substantial gap in modern methods was observed between women with no education (64.9%) and those with secondary education (75.5%; *p* < 0.001). Modern method use was observed considerably high among women who were working (73.8%), those without decision-making autonomy (60.0%), and those exposed to family planning messages (56.3%) (*p* < 0.001). A significant regional variation in modern methods use was evident, with the Central region shown the highest use (55.6%) than their counterparts. Non-use of any method was highest among women aged 35–49 years (33.3%), Muslim (33.1%), those with no education (32.5%), and women in the Northern region (32.5%). Higher non-use was also observed among non-working women (29.3%) and those with four or more living children (27.5%). Although overall traditional method use was low (2.2%), it was considerably high among women in the Northern region (4.3%), those with tertiary education (4.2%), women aged 35–49 years (3.5%), and those in the richest wealth quintile (3.1%).

**Table 4:** Bivariate distribution of current contraceptive use among women aged 15–49 years who desire no more children, Malawi DHS 2015–16 (*N* = 5,358).

Characteristic	Weighted sample (n)	No method (%)	Modern method (%)	Traditional (%)	*p*-value
Age group (years)					
15–19	58	17.0	83.1	0.0	
20–24	471	14.7	85.2	0.1	<0.001
25–34	2,410	19.8	79.0	1.3	
35–49	2,420	33.3	63.2	3.5	
Number of living children					
1 child	231	28.2	71.3	0.5	
2 children	729	20.3	78.7	1.0	<0.001
3 children	1,144	22.1	76.7	1.2	
4 + children	3,254	27.5	69.6	2.9	
Sex composition of living children					
Only girls	510	21.0	76.5	2.5	
Only boys	525	21.5	77.5	1.0	0.003
Both sexes	4,324	26.4	71.4	2.3	
Education level					
No education	864	32.5	64.9	2.6	
Primary	3,430	24.3	73.8	2.0	<0.001
Secondary	961	22.4	75.5	2.1	
Tertiary	103	32	63.8	4.2	
Religion					
Christian	4,693	24.4	73.5	2.1	
Muslim	611	33.1	64.3	2.6	<0.001
Other	54	23.3	76.7	0.0	
Household wealth quintile					
Poorest	1,020	26.1	72.2	1.7	
Poorer	985	24.7	73.9	1.4	
Middle	1,062	25.6	72.4	2.0	<0.001
Richer	1,047	26.5	71.1	2.4	
Richest	1,244	24.3	72.6	3.1	
Employment status					
Not working	1,515	29.3	69.0	1.7	<0.001
Working	3,844	23.9	73.8	2.3	
Decision-making autonomy					
Not autonomous	3,014	23.6	74.7	1.8	<0.001
Autonomous	2,344	27.8	69.6	2.6	
Family planning message exposure					
No exposure	2,948	26.0	71.8	2.2	<0.001
Any media exposure	2,410	24.7	73.3	2.1	
Place of residence					
Urban	1,019	25.5	72.6	2.0	0.804
Rural	4,340	25.4	72.4	2.2	
Region					
Northern	598	32.5	63.3	4.3	
Central	2,131	24.4	74	1.6	<0.001
Southern	2,629	24.6	73.3	2.1	
Total	5,358	25.4	72.4	2.2	

Values are MDHS weighted sample row percentages. Women with limiting fertility demand were included. *p*-values were based on Pearson chi-square tests.

### Predictors of modern and traditional method use among women with fertility limiting demand

Multinomial logistic regression analysis identified several significant associations with modern method use relative to non-use among women with fertility limiting intention (Table 5). Compared with women aged 15–19 years, those aged 25–34 (*β* = −0.912, *p* = 0.020) and 35–49 years (*β* = −1.660, *p* < 0.001) had significantly lower log-odds of modern method use. Educational attainment was positively associated with modern contraceptive use, particularly among women with primary (*β* = 0.204, *p* = 0.024) and secondary education (*β* = 0.252, *p* = 0.048). Women with two (*β* = 0.707, *p* < 0.001), three (*β* = 0.887, *p* < 0.001), and four or more children (*β* = 0.975, *p* < 0.001) had higher log-odds of modern method use compared with women with one child. Employment was positively associated with modern method use (*β* = 0.323, *p* < 0.001), whereas decision-making autonomy was negatively associated (*β* = −0.243, *p* < 0.001). Muslim women had lower log-odds of modern method use compared with Christian women (*β* = −0.428, *p* < 0.001). Regional differences were evident, with women residing in the Central (*β* = 0.429, *p* < 0.001) and Southern regions (*β* = 0.482, *p* < 0.001) showing higher log-odds relative to those in the Northern region. For traditional method use, having four or more children (*β* = 2.239, *p* = 0.033), belonging to the richest wealth quintile (*β* = 1.021, *p* = 0.006), and being employed (*β* = 0.486, *p* = 0.039) were significantly associated with higher log-odds relative to non-use. The interaction results indicate that age and number of living children jointly shape contraceptive behaviour, with older women at lower parities showing reduced likelihood of modern method use, while estimates for traditional methods were unstable due to sparse observations (Supplementary Table).

**Table 5:** Multinomial logistic regression results of adjusted coefficients for modern and traditional contraceptive method use among women aged 15–49 years with a demand for limiting childbearing, Malawi DHS 2015–16 (*N* = 5,358).

Variables	Modern Method *β* Coefficient (SE)	Traditional Method *β* Coefficient (SE)
Age group		
15–19	0.000 (ref.)	0.000 (ref.)
20–24	0.201 (0.210)	10.065 (540.468)
25–34	−0.352 (0.211)	12.223 (540.466)
35–49	−1.291*** (0.215)	12.277 (540.466)
Education level		
No education	0.000 (ref.)	0.000 (ref.)
Primary	0.240*** (0.066)	0.032 (0.261)
Secondary	0.288** (0.094)	0.279 (0.352)
Tertiary	−0.026 (0.190)	0.231 (0.613)
Number of living children		
1 child	0.000 (ref.)	0.000 (ref.)
2 children	0.521*** (0.127)	0.139 (0.745)
3 children	0.920*** (0.137)	0.793 (0.709)
4 + children	1.161*** (0.141)	1.573* (0.708)
Sex composition of children		
Only girls	0.000 (ref.)	0.000 (ref.)
Only boys	−0.086 (0.100)	−0.860 (0.480)
Both sexes	−0.084 (0.086)	−0.747* (0.324)
Household wealth quintile		
Poorest	0.000 (ref.)	0.000 (ref.)
Poorer	0.312*** (0.075)	0.098 (0.367)
Middle	0.370*** (0.075)	0.323 (0.335)
Richer	0.336*** (0.077)	0.600 (0.324)
Richest	0.402*** (0.094)	1.119** (0.355)
Place of residence		
Rural	0.000 (ref.)	0.000 (ref.)
Urban	0.164* (0.082)	−0.087 (0.288)
Region		
Northern	0.000 (ref.)	0.000 (ref.)
Central	0.300*** (0.080)	−0.750** (0.268)
Southern	0.274*** (0.077)	−0.495* (0.248)
Employment status		
Not working	0.000 (ref.)	0.000 (ref.)
Working	0.290*** (0.053)	0.475* (0.224)
Decision-making autonomy		
Not autonomous	0.000 (ref.)	0.000 (ref.)
Autonomous	0.429*** (0.050)	0.940*** (0.190)
Family planning message exposure		
No	0.000 (ref.)	0.000 (ref.)
Yes	0.110* (0.052)	−0.106 (0.204)
Religion		
Christian	0.000 (ref.)	0.000 (ref.)
Muslim	−0.456*** (0.074)	−0.026 (0.289)
Other	0.262 (0.247)	−12.985 (709.174)

Model statistics: Log likelihood = −3,377.217; LR *χ*^2^(46) = 364.04; *p* < 0.001; Pseudo R^2^ = 0.051. Reference outcome category was “No method”. ref.: reference category; AME: average marginal effects. Coefficients were computed with MDHS survey design correction.

Significance levels: *p < 0.05; **p < 0.01; **p < 0.001.

### Reasons for last discontinuation of modern methods

**Figure 2:**
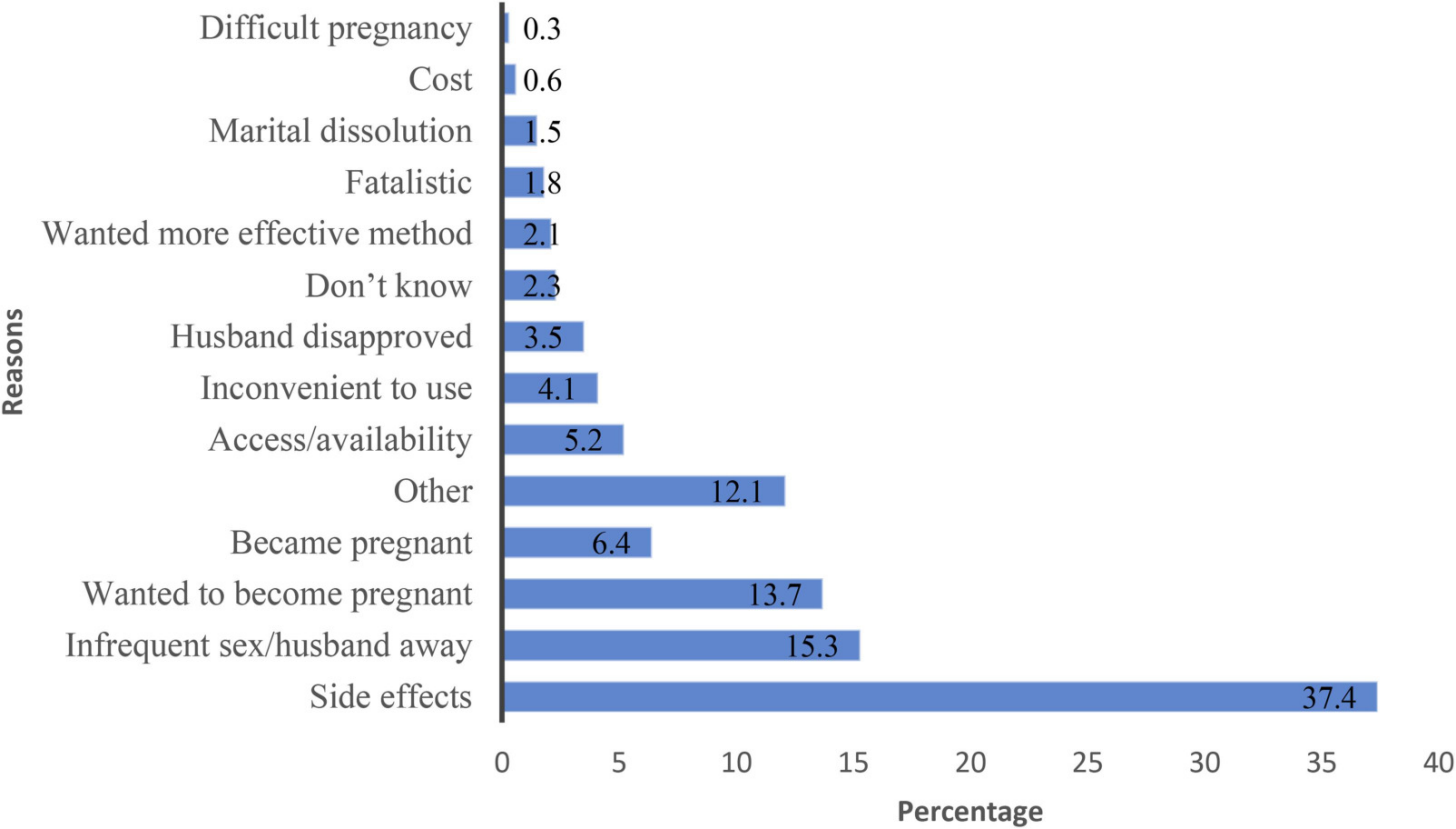
Percentage distribution of reasons for last discontinuation of modern methods among women with demand for limiting childbearing, Malawi DHS 2015–16 (*n* = 655).

Among women with a demand for limiting childbearing who discontinued a modern contraceptive method [Weighted sample (n)= 655], side effects were the most frequently reported reason (37.4%) (Figure 2). Fertility-related reasons were also common, including wanting to become pregnant (13.7%) and becoming pregnant while using a method (6.4%). Infrequent sexual activity or husband's absence accounted for 15.3% of discontinuations. Access-related barriers, such as availability (5.2%), husband's disapproval (3.5%), inconvenience of use (4.1%), and desire for a more effective method (2.1%) were also considerable reasons of last discontinuation.

## Discussion

This study examined fertility intentions and contraceptive behaviour among in-union women with at least one living child in Malawi, offering a refined perspective by focusing on women who have already entered parenthood. Three central findings emerge: (1) parity and age are the strongest predictors of fertility intentions; (2) a substantial proportion of women with limiting demand remain non-users of contraception; and (3) discontinuation of modern methods is primarily driven by side effects rather than access alone.

First, fertility intentions in Malawi are strongly structured by parity and age. The likelihood of wanting additional children declines sharply as the number of living children increases, with particularly large marginal effects among women aged 35–49 years. Similar patterns have been reported in Nigeria, Tanzania, and Kenya, where parity consistently outweighs socioeconomic characteristics in predicting fertility preferences (33, 34). These findings support the theory of adaptive fertility preferences, whereby women revise reproductive goals in response to lived childbearing experiences rather than adhering to fixed pre-marital ideals. In this context, fertility transition appears driven less by abstract ideal family size and more by cumulative reproductive experience, childcare burdens, and economic considerations associated with larger families (35, 36). This finding differs from what many programmes expect, but studies from Rwanda and Senegal report similar weak effects of messaging (28).

Second, despite nearly half of women reporting a desire to stop childbearing, one-quarter of those with limiting demand were not using any contraceptive method. This gap between intention and behaviour echoes findings from studies across sub-Saharan Africa, including Uganda and Ethiopia (37, 38), which document persistent unmet need among women wishing to limit births. While education, wealth, and employment were positively associated with modern contraceptive use in our study, disparities remain evident. Women in higher wealth quintiles and those with secondary education exhibited greater likelihood of modern method use, suggesting that socioeconomic resources facilitate navigation of service barriers, transportation costs, and method-switching options (28). Conversely, poorer and less educated women may face compounded structural barriers, including limited facility access, provider bias, or misinformation regarding side effects.

Religious differences were also observed, with Muslim women more likely to desire additional children and less likely to use modern contraception compared with Christian women. Similar patterns have been documented in the sub-Saharan Africa (39). These differences may reflect variations in community norms, fertility expectations, and gendered decision-making structures rather than doctrinal prohibitions *per se* (40, 41). Importantly, the persistence of these associations after adjustment suggests the need for culturally sensitive, community-engaged family planning approaches use (42). A notable finding is that exposure to family planning messages was not significantly associated with fertility preferences or modern contraceptive use in adjusted models. This aligns with emerging evidence from India indicating that information exposure alone may be insufficient to alter reproductive behaviour when structural and interpersonal constraints remain (30). In settings where awareness of contraception is already high, the binding constraint may no longer be knowledge but concerns about side effects, partner dynamics, or service quality.

Indeed, discontinuation analysis reveals that side effects were the dominant reason for stopping modern methods, followed by infrequent sexual activity and fertility-related reasons. This pattern mirrors DHS-based evidence across sub-Saharan Africa (43), where method-related dissatisfaction is a leading driver of discontinuation. The prominence of side effects suggests gaps in counselling quality, inadequate follow-up support, and limited access to alternative methods. Without effective management of side effects and facilitated switching, women with limiting demand may cycle into non-use, increasing unintended pregnancy risk. Finally, regional differences in contraceptive use indicate persistent geographic inequalities. Women in the Central and Southern regions were more likely to use modern methods compared with those in the Northern region, consistent with spatial analyses of service availability and health infrastructure distribution in Malawi (44). Similar spatial variation in use of modern methods also observed in Ethiopia (45). These differences may reflect uneven distribution of health facilities and outreach services, higher female education and socioeconomic status in more urbanized regions, and variation in cultural norms regarding fertility and contraceptive acceptance. Differences in programmatic intensity, commodity availability, and access to long-acting methods may further widen regional gaps. Together, these structural and socio-cultural factors likely contribute to the observed inequalities.

Overall, the findings suggest that fertility transition is increasingly shaped not by persistent pronatalist preferences but by differential capacity to translate limiting intentions into consistent contraceptive behaviour in Malawi. Addressing this gap requires strengthening service quality, ensuring method choice and switching, reducing socioeconomic barriers, and engaging community and religious leaders to enhance acceptability and informed decision-making.

### Limitations and strengths of the study

Several limitations should be considered when interpreting the findings of this study. First, the cross-sectional design of the Malawi DHS precludes any causal inference. The associations observed between background characteristics, fertility intentions, and contraceptive use reflect correlations at a single point in time and do not establish temporal directionality.

Second, fertility intentions were based on self-reported responses, which may be subject to social desirability bias, recall bias, or ex-post rationalization. Women's stated intentions may also change over time in response to life circumstances, partner dynamics, or economic conditions, potentially affecting the stability of the measured outcome.

Third, contraceptive method classification relied on self-reported current method use. Although DHS employs standardized instruments, misclassification between modern and traditional methods is possible, particularly for fertility awareness-based methods. Such misreporting could introduce measurement error in the outcome variable.

Finally, the analysis was restricted to in-union women with at least one living child and without permanent sterilization. While this restriction strengthens the conceptual focus on women at risk of continued childbearing, it limits the generalizability of findings to unmarried women, formerly married women, or those without children. Therefore, the results should be interpreted within the context of currently married or cohabiting women in Malawi.

Despite these limitations, the study has notable strengths. It applies a refined analytical framework by focusing specifically on women with at least one child and limiting demand, reducing default-response bias. The use of nationally representative DHS data with appropriate sampling weights enhances statistical power, comparability, and policy relevance for Malawi.

### Policy implications

Since fertility intentions and contraceptive behaviour vary strongly by age and number of living children, programs should prioritize older women with multiple children who express limiting demand but exhibit inconsistent contraceptive use. Given that side effects were a leading reason for discontinuation, improving counselling quality, follow-up mechanisms, and method-switching support could enhance sustained contraceptive use among women wishing to stop childbearing. Observed variations by religion and region suggest the need for culturally sensitive, community-engaged interventions that involve religious leaders and male partners to improve acceptability and informed contraceptive choice. Considering disparities by residence and decision-making autonomy, policies should expand access to long-acting reversible contraceptives, strengthen community-based distribution, and integrate empowerment strategies to support women's reproductive decision-making.

## Conclusion

This study shows that fertility intentions among in-union women with at least one child in Malawi are strongly structured by age and parity. The likelihood of wanting additional children declines sharply with increasing number of living children, while uncertainty remains concentrated among specific subgroups. Although most women with limiting demand were using modern contraception, one-quarter were not using any method. Modern method use varied significantly by parity, education, wealth, employment status, religion, and region. Muslim women and older women were less likely to use modern methods, while higher parity and socioeconomic advantage were associated with greater use. Side effects were the most frequently reported reason for discontinuation of modern methods. Overall, the findings indicate that differences in contraceptive behaviour persist even among women who express a desire to stop childbearing. Targeted strategies addressing method continuation, counselling quality, and subgroup disparities are important for improving alignment between fertility intentions and contraceptive practice.

## Data Availability

Publicly available datasets were analyzed in this study. This data can be found here: The data analysed in this study are from the 2015–16 Malawi Demographic and Health Survey (MDHS), available through The DHS Program. The dataset can be requested and downloaded by registered users at The DHS Program data access portal: https://dhsprogram.com/data/available-datasets.cfm.
