# Supplementary material for: From fertility intentions to contraceptive behaviour: evidence among women with children in Malawi
Source: Front Reprod Health. 2026 Apr 22;8:1780852. doi: 10.3389/frph.2026.1780852 (PMC13144022; doi:10.3389/frph.2026.1780852)
Supplement: Supplementary file 1 [file Datasheet1.pdf]

Supplementary File

Table S1 Multicollinearity check using Variance Inflation Factor

	For fertility intention analyses (n=15,866)		For use of contraceptive method analyses (n=5,358)	
**Variable**	**VIF**	**Tolerance**	**VIF**	**Tolerance**
Number of living children	2.777	0.360	2.617	0.382
Age group	2.003	0.499	2.002	0.5
sex composition of children	1.717	0.582	1.726	0.579
Wealth quintile	1.584	0.631	1.582	0.632
Education level	1.42	0.704	1.320	0.758
Residence	1.345	0.744	1.338	0.747
Family planning message exposure	1.156	0.865	1.143	0.875
Region	1.065	0.939	1.051	0.951
Religion	1.057	0.946	1.062	0.942
Employment status	1.033	0.968	1.041	0.96
Decision-making autonomy	1.025	0.976	1.035	0.966
**Mean VIF**	**1.471**		**1.447**	

Note: VIF indicates Variance Inflation Factor

S2. Average Marginal Effects (AMEs) of Interaction Terms on Fertility Preference among Women Aged 15-49 Years, Malawi DHS 2015–16

**Interaction Terms**	**Wants another child AME (SE)**	**p-value**
Age group × Number of living children		
15–19 × 2 children	−0.705*** (0.099)	<0.001
20–24 × 2 children	−0.655*** (0.046)	<0.001
20–24 × 3 children	−0.789*** (0.035)	<0.001
20–24 × 4+ children	−0.873*** (0.045)	<0.001
25–34 × 1 child	−0.320* (0.108)	0.015
25–34 × 2 children	−0.703*** (0.040)	<0.001
25–34 × 3 children	−0.860*** (0.018)	<0.001
25–34 × 4+ children	−0.952*** (0.007)	<0.001
35–49 × 1 child	−0.890*** (0.021)	<0.001
35–49 × 2 children	−0.953*** (0.009)	<0.001
35–49 × 3 children	−0.960*** (0.006)	<0.001
35–49 × 4+ children	−0.988*** (0.002)	<0.001
Education × Residence		
Secondary × Rural	−0.188* (0.074)	0.022
**Interaction Terms**	**Undecided AME (SE)**	**p-value**
Age group × Number of living children		
25–34 × 3 children	−0.532** (0.112)	0.001
25–34 × 4+ children	−0.741*** (0.063)	<0.001

35–49 × 1 child	−0.782** (0.097)	0.001
35–49 × 2 children	−0.650** (0.107)	0.001
35–49 × 3 children	−0.844*** (0.049)	<0.001
35–49 × 4+ children	−0.866*** (0.033)	<0.001
Education × Residence		
Primary × Rural	−0.223* (0.087)	0.024

Note: Values are Average Marginal Effects (AMEs) with standard errors in parentheses from a multinomial logistic regression model. Reference outcome category: *Wants no more children*. Reference interaction categories: women aged 15–19 years with one child and women with no education residing in urban areas. All estimates are adjusted for sex composition of children, household wealth quintile, residence, region, employment status, decision-making autonomy, exposure to family planning messages, and religion. Statistical significance: $p < 0.05$ (*), $p < 0.01$ (**), $p < 0.001$ (***). Only; sparse data and potential quasi-complete separation results are not presented in this table.

Table S3. Multinomial Logistic Regression Results for Interaction Terms Associated with Current Contraceptive Use among Women with Limiting Demand, Malawi DHS 2015–16 (N = 5,350)

**Interaction Terms**	**Modern Method β (SE)**	**Traditional Method β (SE)**
**Age group × Number of living children**		
15–19 × 1 child (ref.)	0.000	0.000
15–19 × 2 children	0.899 (1.147)	0.717 (5541.405)
20–24 × 1 child	−0.499 (0.452)	−0.319 (2652.770)
20–24 × 2 children	0.484 (0.423)	13.345 (2115.257)
20–24 × 3 children	0.762 (0.517)	0.866 (2720.326)
20–24 × 4+ children	0.555 (0.807)	0.842 (4595.695)
25–34 × 1 child	−0.906 (0.494)	−0.786 (2966.978)
25–34 × 2 children	0.014 (0.410)	15.103 (2115.257)
25–34 × 3 children	0.080 (0.398)	15.077 (2115.257)
25–34 × 4+ children	0.027 (0.397)	15.154 (2115.257)
35–49 × 1 child	−1.347** (0.491)	13.849 (2115.257)
35–49 × 2 children	−1.341** (0.431)	13.703 (2115.257)
35–49 × 3 children	−0.887* (0.409)	14.276 (2115.257)
35–49 × 4+ children	−0.598 (0.395)	15.825 (2115.257)
**Education × Place of residence**		
No education × Urban (ref.)	0.000	0.000
No education × Rural	−0.008 (0.325)	15.886 (2037.757)
Primary × Urban	0.250 (0.332)	15.814 (2037.757)
Primary × Rural	0.189 (0.318)	15.777 (2037.757)
Secondary × Urban	−0.037 (0.337)	14.840 (2037.757)
Secondary × Rural	0.398 (0.333)	16.337 (2037.757)
Tertiary × Urban	−0.239 (0.407)	16.403 (2037.757)
Tertiary × Rural	1.130 (0.696)	16.202 (2037.757)

Note: Values are adjusted multinomial logistic regression coefficients (β) with standard errors in parentheses. The base outcome category is No method. Reference categories for interaction terms are: women aged 15–19 years with one living child and women with no education residing in urban areas. All estimates are adjusted for additional covariates included in the full model (sex composition of children, household wealth quintile, region, employment status, decision-making autonomy, exposure to family planning messages, and religion). Statistical significance is denoted as $p < 0.05$ (8), $p < 0.01$ (**), and $p < 0.001$ (***). Very large standard errors in several traditional method interaction estimates indicate sparse data and potential quasi-complete separation; these coefficients should be interpreted with caution
